# Mechanism of the Antigen-Independent Cytokinergic SPE-7 IgE Activation of Human Mast Cells *in Vitro*

**DOI:** 10.1038/srep09538

**Published:** 2015-04-20

**Authors:** Heather J. Bax, Holly Bowen, Tihomir S. Dodev, Brian J. Sutton, Hannah J. Gould

**Affiliations:** 1 Randall Division of Cell and Molecular Biophysics, King's College London, London, United Kingdom; 2 MRC & Asthma UK Centre in Allergic Mechanisms of Asthma, London, United Kingdom; 3 NIHR Biomedical Research Centre at Guy's and St Thomas' NHS Foundation Trust and King's College London, London, United Kingdom

## Abstract

Release of pro-inflammatory mediators by mast cells is a key feature of allergic disease. The ‘dogma’ is that IgE molecules merely sensitise mast cells by binding FcεRI prior to cross-linking by multivalent allergen, receptor aggregation and mast cell activation. However, certain monoclonal IgE antibodies have been shown to elicit mast cell activation in an antigen-independent cytokinergic manner, and DNP-specific murine SPE-7 IgE is the most highly cytokinergic antibody known. We show that both monovalent hapten and recombinant SPE-7 IgE Fab inhibit its cytokinergic activity as measured by mast cell degranulation and TNF-α release. Using SPE-7 IgE, a non-cytokinergic human IgE and a poorly cytokinergic murine IgE, we reveal that interaction of the Fab region of ‘free’ SPE-7 IgE with the Fab of FcεRI-bound SPE-7 IgE is the basis of its cytokinergic activity. We rule out involvement of IgE Fc, Cε1 and Cλ/κ domains, and propose that ‘free’ SPE-7 IgE binds to FcεRI-bound SPE-7 IgE by an Fv-Fv interaction. Initial formation of a tri-molecular complex (one ‘free’ IgE molecule cross-linking two receptor-bound IgE molecules) leads to capture of further ‘free’ and receptor-bound IgEs to form larger clusters that trigger mast cell activation.

IgE plays a critical role in mast cell mediated type I hypersensitivity in allergic disease. The ‘dogma’ of mast cell activation is that IgE bound to its high-affinity receptor, FcεRI, must be cross-linked by multivalent antigen (allergen) to cause receptor aggregation, signal transduction and the release of pro-inflammatory mediators that initiate the allergic response^1^,^2^,^3^. However, it has been shown that antigen is not required for certain monomeric IgE antibodies to elicit activation of mast cells^4^,^5^,^6^,^7^,^8^,^9^,^10^,^11^,^12^,^13^,^14^,^15^,^16^,^17^,^18^,^19^,^20^,^21^,^22^,^23^. These IgE antibodies, and the activity that they exhibit, were termed cytokinergic by Kitaura and colleagues over ten years ago^10^.

The DNP-specific murine IgE, SPE-7, is the most highly cytokinergic antibody known, inducing mast cell survival, migration, fibronectin adhesion, FcεRI upregulation, cytokine release and degranulation in the absence of antigen^8^,^10^,^15^,^20^,^22^,^23^. However, the mechanism by which SPE-7 IgE and other cytokinergic IgE antibodies elicit some or all of these activities, the structural determinants required for these activities, and crucially the implications for human allergic disease, are unknown.

Kitaura and co-workers ranked a number of murine IgEs, from the most poorly to the most highly cytokinergic IgEs, on the basis of their ability to perform an increasing number cytokinergic activities and also the strength of these activities^10^. SPE-7 IgE has proved to be the most highly cytokinergic IgE and the most widely adopted for mechanistic studies. A number of features are associated with the cytokinergic activity of SPE-7 IgE and other highly cytokinergic IgEs. Firstly, as with antigen activation of IgE-sensitised mast cells, aggregation of FcεRI on the surface of mast cells was observed upon stimulation with highly cytokinergic IgEs, including SPE-7 IgE^8^,^10^. Secondly, a 100-fold greater concentration of these IgEs (1–5 μg/ml), compared to the range of concentrations required for the sensitisation of mast cells for antigen activation, is required for cytokinergic activity. Thirdly, removal of the ‘free’ IgE, that was not bound tightly to FcεRI on mast cells resulted in ablation of the cytokinergic activity, while its replacement restored the ability to trigger cell activation in the absence of antigen, implicating ‘free’ IgE in the mechanism^7^,^15^. Finally, the available evidence suggests that IgE variable regions are important for cytokinergic activity. Kitaura *et al*. reported that murine IgEs differing only in their VH and/or VL domain sequences elicit a wide range of cytokinergic activities^10^. Furthermore, it was shown that monovalent antigen inhibits the activity of cytokinergic IgE^10^,^15^. Thus, it appears that antigen binding may interfere with a critical interaction between the ‘free’ IgE molecules in solution and a site on the mast cell surface, culminating in cytokinergic activity.

In the present study we focus on the mechanism of cytokinergic SPE-7 IgE activity in a reproducible human system using the LAD-2 human mast cell line. We demonstrate that some of the important features observed in the rodent system hold true in this human system, including the critical role of the Fv region of SPE-7 IgE. We also demonstrate for the first time that the cytokinergic activity of SPE-7 IgE requires both the saturation of FcεRI on the LAD-2 cells by SPE-7 IgE and the presence of ‘free’ SPE-7 IgE in solution. We therefore propose a mechanism by which the Fv regions of ‘free’ SPE-7 IgE molecules cross-link the Fv regions of adjacent receptor-bound SPE-7 IgE molecules to induce mast cell activation.

## Results

### A reproducible human system to study the mechanism of cytokinergic IgE activity

Human IgE does not bind to murine FcεRI, but paradoxically both human and murine IgE bind to human FcεRI^24^. Previous work has demonstrated that certain human myeloma IgEs displayed cytokinergic IgE activity *in vitro* when incubated with cord blood or human lung primary mast cells^9^,^18^,^19^,^21^,^25^. We replicated this work in peripheral blood primary mast cells, but found this system gave results that were highly variable between donors. We therefore developed the LAD-2 human mast cell line system for the present experiments. This system required shorter priming periods than primary cells and eliminated donor variability.

**Figure 1:**
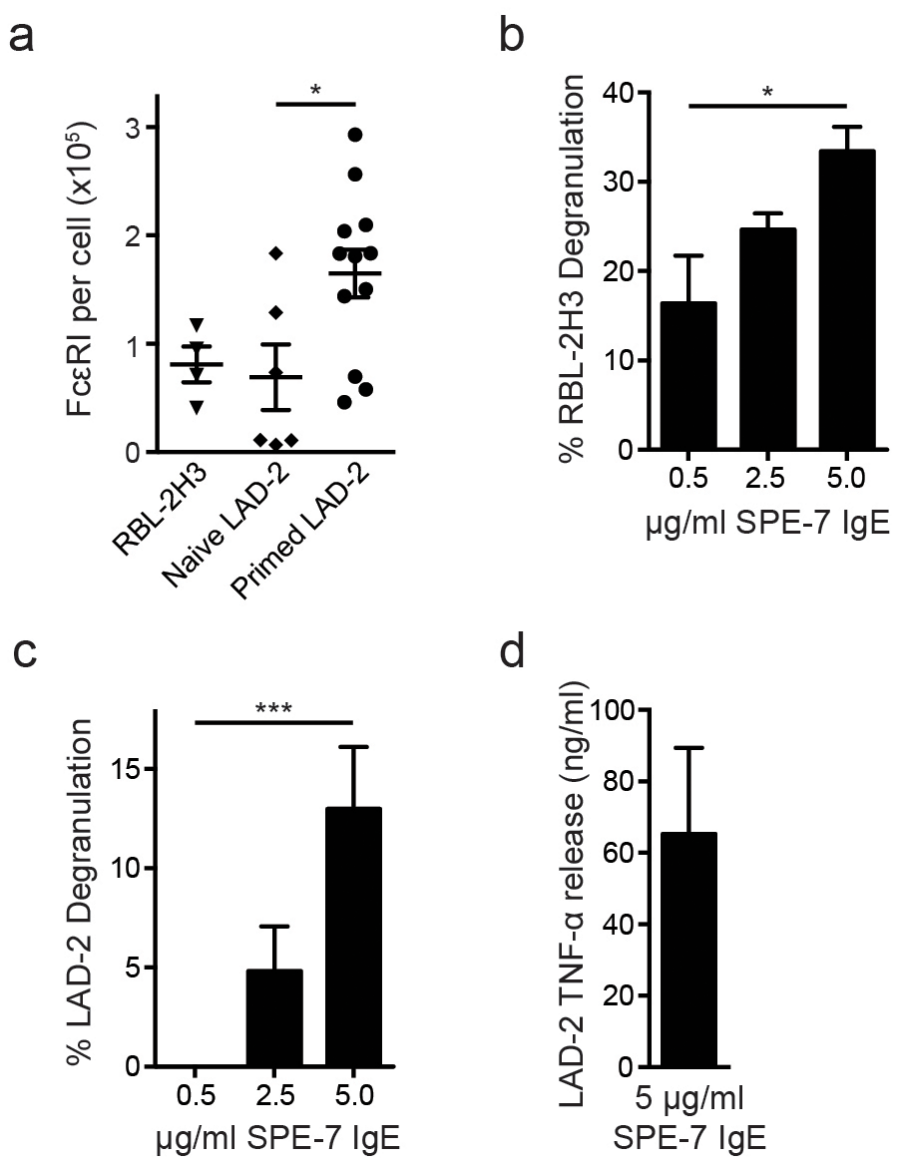
Rat and human mast cell systems and activation by highly cytokinergic SPE-7 IgE. (A) The number of rat FcεRI molecules expressed per RBL-2H3 and human FcεRI molecules expressed per LAD-2 mast cells were quantified by Qifikit® (Dako). RBL-2H3 cells express 0.8 ± 0.2 × 10^5^ rat FcεRI molecules per cell and naïve LAD-2 cells and those primed with 6 ng/ml IL-4 for 5 days express 0.7 ± 0.3 × 10^5^ and 1.7 ± 0.2 × 10^5^ human FcεRI molecules per cell, respectively (n = 4, 6, and 12, respectively). (B) RBL-2H3 degranulation (over 14% baseline; n = 3–6), (C) LAD-2 mast cell degranulation (over 9% baseline; n = 7), and (D) LAD-2 TNF-α release (over 42 ng/ml baseline; n = 7), evoked by highly cytokinergic SPE-7 IgE. All data are shown as mean ± SEM. Statistically significant difference was determined by one-way ANOVA with Tukey's post-test; *** p < 0.001, * p = 0.01 to 0.05

We first quantified the level of receptor expression relative to the RBL-2H3 rat basophilic cell line, often used in studies of murine cytokinergic IgEs. To compare the levels of FcεRI on the LAD-2 and RBL-2H3 cells we used a quantitative flow cytometric assay calibrated with beads bearing precisely known numbers of ligands. RBL-2H3 cells expressed a mean ± SEM of 0.8 ± 0.2 × 10^5^ rat FcεRI molecules per cell, similar to the level of receptor expressed by naïve LAD-2 cells (mean ± SEM of 0.7 ± 0.3 × 10^5^ human FcεRI per cell), which increased to 1.7 ± 0.2 × 10^5^ upon addition of 6 ng/ml IL-4 to the cell culture for 5 days prior to receptor quantification (Figure 1A).

As previously reported^15^, RBL-2H3 cells were activated, in the absence of antigen, by SPE-7 IgE (Figure 1B). Similarly, degranulation and TNF-α secretion of IL-4 primed LAD-2 mast cells by SPE-7 IgE was observed (Figures 1C and D), indicating for the first time, the antigen-independent activation of human mast cells by this highly cytokinergic murine IgE. Upon comparison with poorly and moderately cytokinergic IgEs, as judged previously in the rodent system, SPE-7 IgE maintained its superior ranking in the level of cytokinergic activity using the human LAD-2 cell line degranulation assay (data not shown).

### Monovalent DNP-L-serine inhibits the cytokinergic activity of SPE-7 IgE in human mast cells

**Figure 2:**
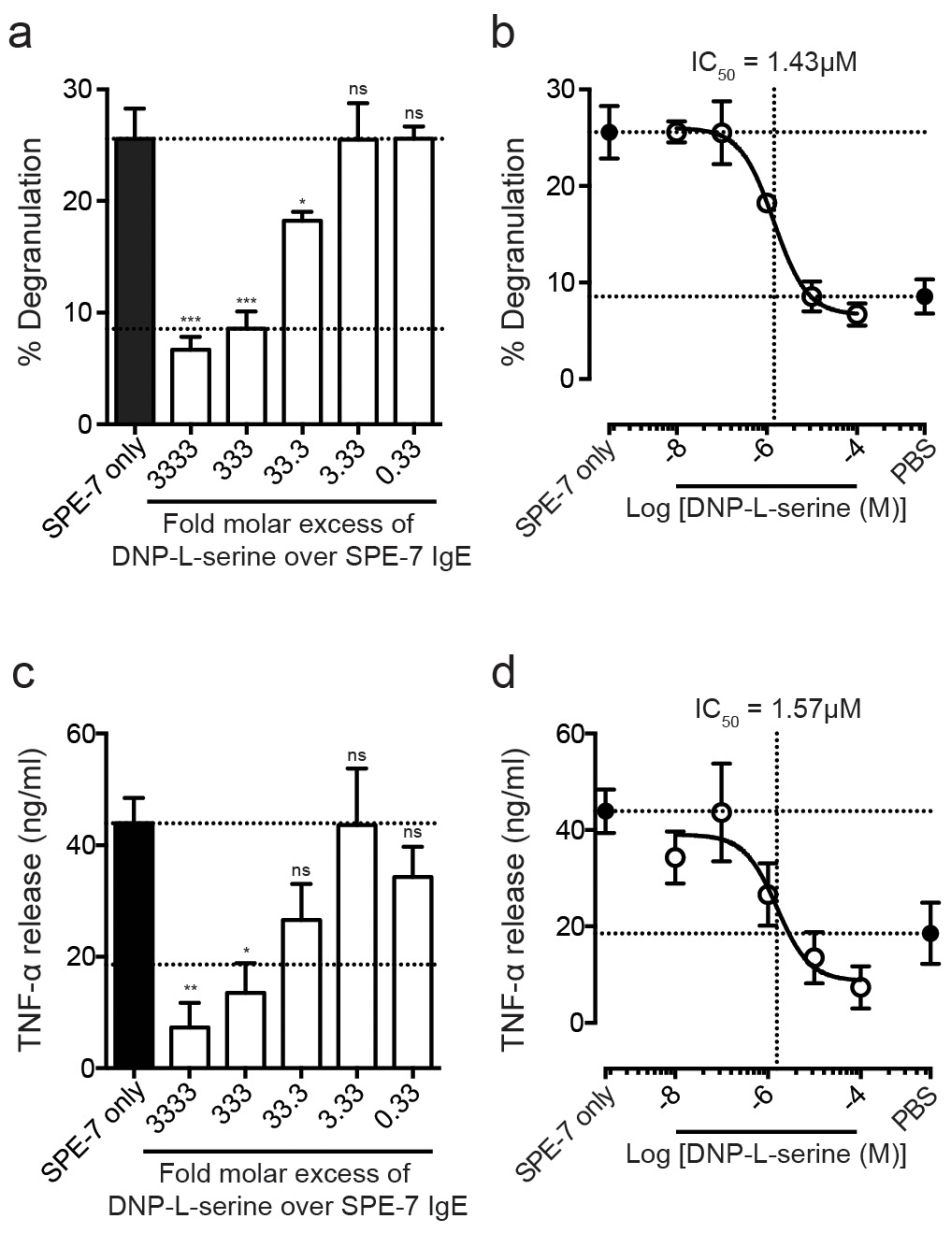
Monovalent DNP inhibits SPE-7 IgE induced LAD-2 cell activation. (A) Percentage degranulation of LAD-2 cells, induced by 30 nM SPE-7 IgE, was dose-dependently inhibited by addition of indicated molar excess of monovalent DNP-L-serine. (B) Data are represented as an inhibition curve. IC_50_ of monovalent DNP-L-serine inhibition of SPE-7 IgE induced degranulation = 1.43 μM. (C) LAD-2 cell TNF-α release, induced by 30 nM SPE-7 IgE, was dose-dependently inhibited by addition of indicated molar excess of monovalent DNP-L-serine. (D) IC_50_ of monovalent DNP-L-serine inhibition of SPE-7 IgE induced TNF-α release = 1.57 μM. Lower and upper dashed lines indicate cell-only background control and activation by SPE-7 IgE only, respectively. Means of 3 to 7 independent experiments ± SEM are shown. Statistically significant difference to SPE-7 IgE only was determined by one-way ANOVA with Dunnett's post-test; *** p < 0.001, ** p = 0.001 to 0.01, * p = 0.01 to 0.05, ns = not significant p ≥ 0.05.

Different murine IgE antibodies activate mast cells in a cytokinergic manner to varying degrees, implicating the variable region in cytokinergic IgE activity^7^,^8^,^10^. Consistent with this hypothesis, as shown previously in rodent systems, we observed the dose-dependent inhibition of SPE-7 IgE-mediated degranulation of LAD-2 human mast cells by the monovalent antigen DNP-L-serine (Figure 2A). Degranulation was completely ablated in the presence of a 333-fold molar excess (10 μM) of the monovalent antigen. Plotting these data as an inhibition curve (Figure 2B) shows an IC_50_ value of 1.43 μM. This was accompanied by the dose-dependent inhibition of SPE-7 IgE-mediated TNF-α release in the same monovalent antigen concentration range (Figure 2C) with a similar IC_50_ value of 1.57 μM (Figure 2D). These data are strikingly similar to the results of Kitaura *et al*. and Pandey *et al.*, in which an IC_50_ value of 2.00 μM for inhibition of IL-6 secretion by bone marrow-derived murine mast cells^10^ and 100% inhibition of RBL-2H3 cell degranulation by 11 μM DNP-serine^15^ were reported.

### Recombinant SPE-7 IgE Fab inhibits the mast cell activation induced by the SPE-7 IgE

**Figure 3:**
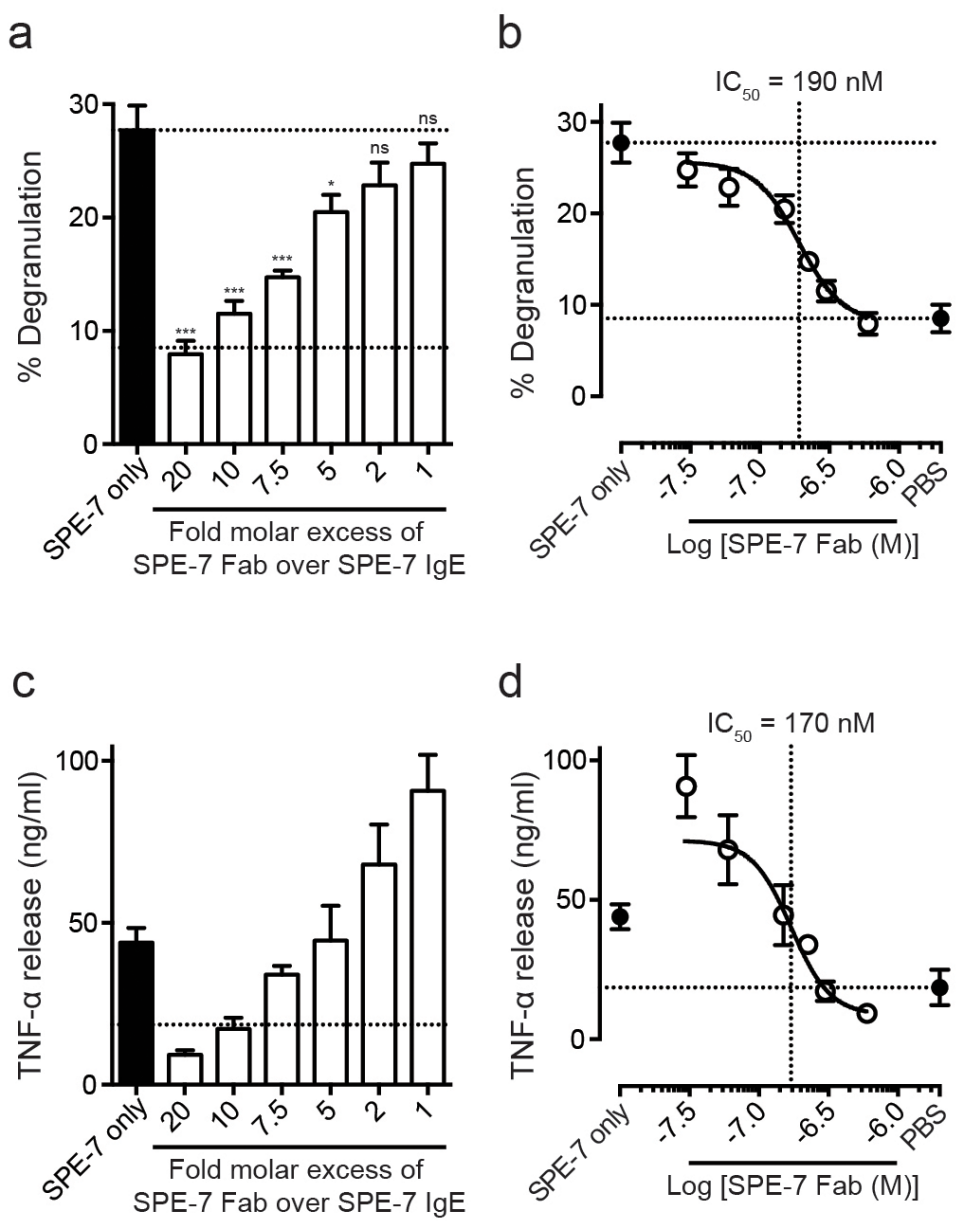
SPE-7 IgE Fab inhibits SPE-7 IgE induced LAD-2 cell activation. (A) Percentage degranulation of LAD-2 cells, induced by 30 nM SPE-7 IgE, was dose-dependently inhibited by addition of indicated molar excess of recombinant SPE-7 IgE Fab. (B) Data are shown in an inhibition curve. IC_50_ of SPE-7 IgE Fab inhibition of SPE-7 IgE induced degranulation = 190 nM. Lower and upper dashed lines indicated cell-only background control and activation by SPE-7 IgE only, respectively. (C) LAD-2 cell TNF-α release, evoked by 30 nM SPE-7 IgE, was dose-dependently inhibited by addition of indicated molar excess of recombinant SPE-7 IgE Fab. (D) IC_50_ of recombinant SPE-7 IgE Fab inhibition of SPE-7 IgE evoked TNF-α release ~170 nM. Dashed line indicates cell-only background control. Means of 4 to 10 independent experiments ± SEM are shown. Statistically significant difference to SPE-7 IgE only was determined by one-way ANOVA with Dunnett's post-test; *** p < 0.001, * p = 0.01 to 0.05, ns = not significant p ≥ 0.05.

Inhibition of the cytokinergic activity by monovalent DNP-L-serine (Figure 2) suggests that blockade of the antigen binding site in the Fab region of SPE-7 IgE impedes the cytokinergic activity. We therefore generated a monovalent recombinant SPE-7 IgE Fab (Supplementary Table and Supplementary Figures 1 and 2) to investigate the role of the Fab region of IgE in the mechanism of cytokinergic activity. Addition of a range of molar excess concentrations of recombinant SPE-7 IgE Fab over SPE-7 IgE resulted in the dose-dependent inhibition of LAD-2 human mast cell degranulation and TNF-α release (Figures 3A and C) with IC_50_ values of 190 nM and 170 nM, respectively (Figures 3B and D). Thus recombinant SPE-7 IgE Fab is ~8-fold more potent in its inhibition of SPE-7 IgE cytokinergic activity than monovalent DNP (180 nM vs. 1.50 μM).

### Fv-Fv-mediated interaction of ‘free’ with receptor-bound SPE-7 IgE

**Figure 4:**
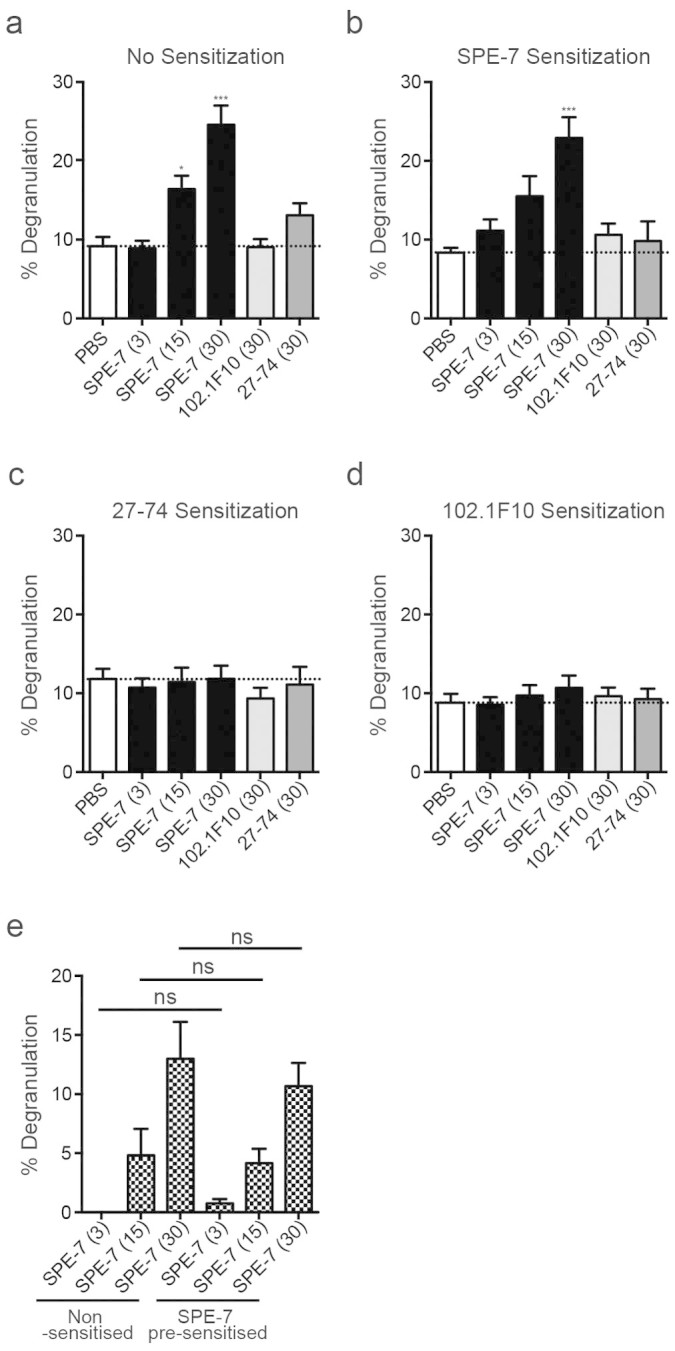
Human Mast Cells are Activated by a Combination of FcεRI-Bound and ‘Free’ SPE-7 IgE. Percentage degranulation of LAD-2 cells, induced by indicated nM concentration of SPE-7, 102.1F10 and 27–74 IgE antibodies, in non-sensitised cells (A, n = 2–6) and those sensitised with SPE-7 IgE (B, n = 2–5), 27–74 IgE (C, n = 2–3) or 102.1F10 IgE (D, n = 2–6). Statistically significant difference to PBS background control was determined by one-way ANOVA with Dunnett's post-test; *** p < 0.001, ** p = 0.001 to 0.01. Comparison of degranulation in non-sensitised cells or cells pre-sensitised with SPE-7 IgE (E). Statistically significant difference was analysed by one-way ANOVA with Tukey's post-test. All data are shown as mean ± SEM.

Earlier studies in the rodent system showed that removal of ‘free’ IgE ablates cytokinergic mast cell activation^7^,^15^. We therefore investigated the necessity for SPE-7 IgE to be bound to FcεRI and/or be ‘free’ in solution. LAD-2 human cells that were primed with IL-4 and stem cell factor alone, or in combination with an FcεRI-saturating concentration of SPE-7 IgE below the threshold (6 nM; 1 μg/ml) for cytokinergic activity, were activated by addition of increasing concentrations of SPE-7 IgE (Figures 4A and B). There was no significant difference between the cytokinergic activity elicited by SPE-7 IgE, whether or not cells were pre-sensitised with SPE-7 IgE (Figures 4A, B and E). However, cells that had been sensitised and FcεRI-saturated with either a poorly cytokinergic murine IgE 27–74 (Figure 4C), non-cytokinergic human IgE 102.1F10 (Figure 4D), or human IgE Fcε_2–4_ (constant-region fragment, data not shown), were not activated upon addition of SPE-7 IgE, even at 30 nM (5 μg/ml). The non-cytokinergic and poorly cytokinergic nature of 102.1F10 and 27–74 IgE antibodies, respectively, were demonstrated in non-sensitised LAD-2 human mast cells, primed with IL-4 and stem cell factor alone, and in all sensitisation conditions (Figures 4A–D). These studies indicate that SPE-7 IgE must bind FcεRI and also be ‘free’ in solution, and that either of these IgEs alone is insufficient for cytokinergic activity. The data also show that ‘free’ SPE-7 IgE does not act by directly cross-linking non-sensitised FcεRI, as observed for anti-FcεRI IgG antibodies^26^,^27^,^28^. If this were the case mast cell activation would be observed regardless of the IgE used to saturate the receptor.

Our data also rule out a role for the Fc region of either ‘free’ or receptor-bound IgE. Addition of non-cytokinergic human IgE 102.1F10 (with human Fc) or poorly-cytokinergic murine IgE 27–74 (with murine Fc) did not activate cells sensitised with SPE-7 IgE (Figure 4B). Furthermore, the addition of SPE-7 IgE did not activate cells sensitised with either 102.1F10 or 27–74 IgE antibodies (Figures 4C and D). By the same token, this argument rules out the involvement of the Cε1 domain, human or murine, and the constant λ or κ domains, as these domains are present in 102.1F10 and 27–74 IgEs, respectively. This implies that the interaction between ‘free’ and receptor-bound SPE-7 IgE occurs exclusively through the Fv regions.

## Discussion

In the present work we have demonstrated that human mast cells of the LAD-2 cell line can be activated by highly cytokinergic murine SPE-7 IgE (Figures 1B and C). Thus LAD-2 cells can provide a reproducible human system with which to explore the mechanism of cytokinergic IgE activity. We report that SPE-7 IgE activates human mast cell degranulation and cytokine release in a Fab-dependent manner (Figures 2 and 3). The importance of the variable regions of cytokinergic IgE was first suggested when it was observed that murine IgEs, all with murine constant domains, but with differing heavy- and/or light-chain variable domains, elicit cytokinergic activity to varying degrees within a hierarchy of such activities^7^,^8^,^10^. This was originally supported by the inhibition of the cytokinergic activity of DNP- and TNP-specific IgE by monovalent DNP and TNP, respectively^10^,^15^. Our studies using human mast cells are in agreement with these observations (Figure 2).

The inhibition of SPE-7 IgE-dependent mast cell degranulation and cytokine release by recombinant SPE-7 IgE Fab in the present study (Figure 3) further demonstrates that the Fab competes with the free IgE for binding to receptor-bound IgE. Unlike whole IgE, SPE-7 IgE Fab is monomeric and thus lacks the ability to cross-link and thereby induce the clustering of receptors required for antigen activation of mast cells by cytokinergic IgE. The inhibitory activity of the SPE-7 IgE Fab is ~8-fold greater than that of the monomeric antigen (DNP-L-serine), perhaps suggesting that the antigen-binding site is only part of the critical site of activation, or that antigen binding changes the conformation of SPE-7 IgE so that this site is masked. SPE-7 IgE is notoriously polyreactive and different antigens are known to bind to different conformations of the variable domain^29^,^30^,^31^,^32^. Thus the ‘epitopes’ recognised in the Fab-Fab interaction remain undefined.

In the light of these results, the necessity for cytokinergic IgE to be bound via its Fc to FcεRI and/or to be ‘free’ in solution was then studied. Two previous studies in rodent systems have shown that the presence of ‘free’ IgE is required for cytokinergic activity. Asai and co-workers demonstrated that increased survival of murine BMMCs was immediately ablated on removal of ‘free’ IgE, and Pandey *et al.* showed that calcium flux in RBL-2H3 cells was significantly reduced upon removal of ‘free’ SPE-7 IgE by washing^7^,^15^. As expected, owing to the long half-life (16 hours) of the IgE-FcεRI complex on cells in suspension, these quiescent cells remained sensitised with receptor-bound IgE, as subsequent addition of multivalent antigen resulted in mast cell activation. The activity of ‘free’ IgE was implicated, as experiments showed that quiescent IgE-sensitised cells were also activated upon reconstitution with ‘free’ IgE, in the absence of antigen^15^. This result is also consistent with the requirement for extremely high concentrations of cytokinergic IgE to induce mast cell activation.

**Figure 5:**
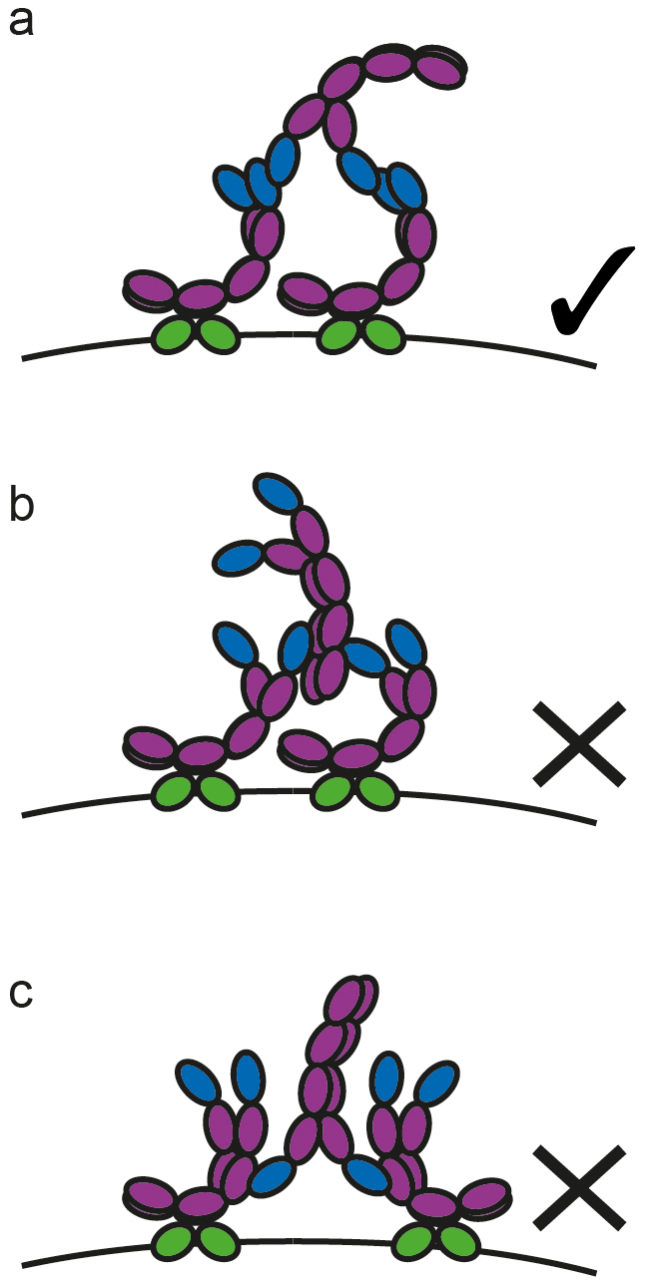
Proposed Mechanism of the Cytokinergic Activity of SPE-7 IgE. Complexes may be formed by ‘free’ SPE-7 IgE bridging FcεRI-bound SPE-7 IgE in an Fv-Fv manner (A). Interactions between the Fv region of receptor-bound IgE and Fc of ‘free’ IgE (B) or between the Fc of receptor-bound IgE and Fv regions of ‘free’ IgE (C) have been ruled out. Variable and constant IgE domains are shown in blue and purple, respectively. Extracellular FcεRIα subunits are shown in green. The cytoplasmic region of the α-chain, and the β- and γ-chains of FcεRI have been omitted for clarity.

It was not clear from this earlier work whether FcεRI-bound IgE and ‘free’ IgE must both be cytokinergic for activation of the mast cells. We have now shown that mast cells must be sensitised with SPE-7 IgE and stimulated with high concentrations of ‘free’ SPE-7 IgE to trigger degranulation in the absence of antigen (Figure 4). We suggest that addition of SPE-7 IgE first results in saturation of the FcεRI receptors due to the high affinity of this interaction (KD ~10^−10^ M), followed by the capture of ‘free’ IgE by a Fab-Fab interaction to form a tri-molecular complex (Figure 5A). The capture of further molecules of ‘free’ IgE may add to this complex. This additional cross-linking may bring in other receptor-bound IgEs, thus forming sufficiently large clusters of IgE-FcεRI complexes to trigger mast cell activation.

In this view of the mechanism we have been able to rule out interactions between the Fab region of receptor-bound IgE and Fc of ‘free’ IgE (Figure 5B) or conversely between the Fab of ‘free’ IgE and Fc of receptor-bound IgE (Figure 5C) by using combinations of highly-cytokinergic SPE-7 IgE and other non- or poorly cytokinergic IgEs for sensitisation and activation. The non-cytokinergic IgE antibody 102.1F10 has human Fc, while the poorly cytokinergic 27–74 IgE has murine Fc. Thus the experiments that involve sensitising and challenging with all combinations of these two antibodies together with SPE-7 IgE (Figure 4) rule out involvement of the Fc regions of both species. Similarly we can rule out the involvement of the Cε1 domains. Furthermore, since IgE 102.1F10 has a λ light chain, and IgE 27–74 a κ chain, the Cλ and Cκ domains can also be excluded. Thus we conclude that the interaction between ‘free’ and receptor-bound SPE-7 occurs through the Fv regions (as depicted in Figure 5A).

The ability of a cytokinergic IgE to form Fv-Fv interactions may also be due to inherent conformational lability and polyreactivity of SPE-7 IgE. James and colleagues revealed the conformational isomerism of SPE-7 IgE by structural studies, where the predominant conformer Ab^1^ (PDB ID: 1OAQ) was observed in 80% of SPE-7 molecules^30^. This conformer has a broad and flat surface at the CDRH3 pocket and binds proteins, such as thioredoxin, to form conformer Ab^4^ (PDB ID: 1OAZ). In contrast, the Ab^2^ conformer (PDB ID: 1OCW) was seen in around 20% of SPE-7 molecules, and has a deep pocket, which binds a wide range of haptens, such as alizarin red and furazolidone (PDB ID: 1OAR and 1OAY, respectively), with a range of affinities (20 nM to 100 μM). Furthermore, upon binding of the higher affinity hapten DNP-lysine (KD = 2 × 10^−8^ M), the SPE-7 IgE Fab domain undergoes induced fit rearrangement to conformer Ab^3^ (PDB: ID 1OAU)^29^,^30^,^33^. Similarly, in ELISA screens of IgE binding to autoantigens, Kashiwakura and colleagues observed experimentally the polyreactivity inferred from such multiple conformational states^32^. SPE-7 IgE exhibited polyreactivity to three of the five autoantigens tested: single- and double- stranded DNA and thyroglobulin, but not β-galactosidase or histamine-releasing factor (HRF). By screening a range of IgE antibodies, these experiments demonstrated that highly cytokinergic IgE antibodies were more polyreactive than poorly cytokinergic, thus relating these two properties in IgE.

There are examples of Fv-mediated self-association in other immunoglobulin classes, and crystallographic studies have shown a number of potential modes of self-association. Boehm *et al.* showed interactions between VH and VL domains of two single chain Fvs (scFv) of MFE-23, and Calarese and colleagues demonstrated VH domain swapping between neighbouring anti-HIV 2G12 Fabs^31^,^34^,^35^. Although it is not clear which specific structural determinants of these antibodies are responsible for the self-association, they are all Fv-dependent, which is consistent with our findings that the interaction is Fv-Fv mediated and without involvement of the Cε1 or Cλ/κ domains.

It is unlikely that at the concentrations used in these experiments, or those that occur physiologically, IgE would self-associate without first being concentrated by binding to receptors on the cell surface. Binding affinities are increased by many orders of magnitude when reactants are confined to the plane of a cell membrane, due to increased local concentration brought about by significantly reduced orientational freedom and the constraint to move laterally within the membrane (in two compared to three dimensions in solution). DeLisi demonstrated this by calculating a reduction in KD by a factor of 10^5^ when compared to the same reactants in solution^36^. Similarly, it has been calculated that with 3 × 10^5^ FcεRI molecules per cell, which is close to the mean ± SEM of 1.7 ± 0.2 × 10^5^ FcεRI molecules per LAD-2 human mast cell or RBL-2H3 cell measured in our work (Figure 1A), there would be a high probability of collisions^37^. Furthermore, the orientations of the Fabs have been modelled for IgE bound to FcεRI and they appear to be accessible for ‘free’ IgE binding^31^,^38^, thus cross-linking of IgE-receptor complexes by the Fv regions of ‘free’ IgE molecules is plausible.

We attempted to detect Fv-Fv mediated interactions between SPE-7 IgE molecules and/or recombinant SPE-7 IgE Fab by ELISA, surface plasmon resonance (SPR) and flow cytometry (data not shown). Despite a high density of molecules on the SPR surface and the same high concentrations of SPE-7 IgE and SPE-7 IgE Fab as in the activation assays, we were unable to detect interactions between the molecules. This probably reflects the low affinity of an individual Fv-Fv mediated interaction between receptor-bound and ‘free’ cytokinergic IgE molecules, and explains why extremely high concentrations of IgE are required to manifest the avidity of the interaction between ‘free’ IgE and receptor-bound IgE on the mast cell membrane.

We have demonstrated an Fv-Fv mediated mechanism for the cytokinergic activity of SPE-7 IgE. Our study has made use of purified recombinant Fabs, and a similar approach could be extended to locate the interaction sites in the ‘free’ and bound IgEs more precisely. Exchange of heavy and light chains between recombinant IgEs would establish whether only one or both chains in SPE-7 IgE are required to form these sites, and mutagenesis or structural studies such as X-ray crystallography or NMR could be applied to determine the epitopes involved. Currently we do not know whether the mechanisms or locations of the binding sites are the same for cytokinergic IgEs other than SPE-7 IgE.

A continuing effort to understand the cytokinergic activity of IgE is important because this activity may well be involved in the pathogenesis of chronic diseases associated with IgE, including eczema, allergic rhinitis, asthma, and food allergies. Cytokinergic IgE may be involved in both atopic and non-atopic (where no allergenic stimulus has been identified) forms of these diseases. However, it is not yet known what effect cytokinergic IgE may have on other FcεRI- and CD23-expressing cells^31^. Recent studies in humanised mice have reported that IgE–FcεRI binding on dendritic cells and monocytes may result in receptor internalisation and contribute to serum IgE clearance^39^ and that signals from antigen cross-linking of IgE-FcεRI complexes on dendritic cells may restrain allergic inflammation^40^. It may therefore be possible that cytokinergic IgE also acts in a regulatory, as well an inflammatory manner. Nevertheless, as discussed by the authors of these studies, a regulatory role of IgE is clearly not sufficient to resolve the uncontrolled immune response in inflammatory disease^40^.

Our conclusion that Fv-Fv interaction is the basis for cytokinergic activity does not contradict the ‘dogma’ that cross-linking is required for receptor aggregation and mast cell activation^1^,^2^,^3^,^37^. In fact, our conclusions strengthen this paradigm, demonstrating that receptor cross-linking is required either by binding of multivalent antigen to IgE-FcεRI complexes, or by the Fv-Fv interaction between ‘free’ and FcεRI-bound cytokinergic IgE molecules. The mechanism of cytokinergic activity is also reminiscent of the phenomenon of idiotype/anti-idiotype (Id/anti-Id) antibody recognition. Although the Id/anti-Id paradigm has been proposed as a tolerogenic mechanism for regulating antibody production^41^,^42^, in the context of an IgE anti-IgE interaction, this may well have pathogenic consequences in allergic disease.

## Methods

### SPE-7 cDNA synthesis and sequencing

SPE-7 IgE hybridoma cells (NS1 cells; kindly provided by Prof. Zelig Eshhar^43^) were lysed and RNA was extracted using an RNeasy mini kit (Qiagen). cDNA was generated with Maxima® reverse transcriptase (Fermentas).

Heavy- and light-chain for SPE-7 IgE were amplified from hybridoma cDNA by PCR using Phusion High Fidelity DNA polymerase (Finnzymes). Light-chain and heavy chain forward and reverse primers (see Supplementary Table) were designed based on murine antibody mAb9B11 lambda light-chain (GenBank accession no. EF392840) and the SPE-7 IgE sequence reported by James and Tawfik and the murine IgE constant domains sequence reported by Shinkai et al., respectively^33^,^44^. PCR reactions were carried out as follows: 98°C for 30 seconds initial denaturation, 30 cycles of 98°C denaturation for 10 seconds, annealing at 55°C for 30 seconds, extension at 72°C for 45 seconds, and final extension at 72°C for 5 minutes. NS1 hybridoma cell-derived SPE-7 IgE Fab heavy- and light-chain sequences are deposited in GenBank (Accession nos. KJ734989 and KJ734990; Supplementary Figure 1).

### Fab PIPE Cloning

Polymerase incomplete primer extension (PIPE) cloning^45^ was utilised to clone a murine SPE-7 IgE Fab. pVITRO1 vector (Invivogen), was first linearised at MCS1 by BspEI and AvrII restriction enzyme digest (New England Biolabs). In parallel, SPE-7 IgE light-chain was amplified with Phusion High Fidelity DNA polymerase (Finnzymes), using forward and reverse primers to add regions homologous to the digested pVITRO vector (Supplementary Table). PCR reaction was carried out as above with an annealing temperature of 61°C and no final extension. Recombination of linearised pVITRO vector and SPE-7 IgE light chain insert by PIPE cloning was carried out as previously described^46^.

This purified pVITRO1_SPE-7 IgE light-chain plasmid was linearised at MCS2 by AgeI and NheI restriction enzyme digest (New England Biolabs). SPE-7 IgE Fab heavy-chain (comprising the SPE-7 IgE VH-region coupled to the Cε1 constant domain) was amplified and a 6×-histidine tag added at the N-terminus using round 1 PCR primers, followed by a second round of PCR to add regions homologous to linearised pVITRO1_SPE-7 IgE light-chain plasmid. Both rounds of PCR were set up and carried out as for the light-chain (see Supplementary Table for primers) and PIPE cloning^46^ used to combine SPE-7 IgE Fab heavy-chain and linearised pVITRO1_SPE-7 IgE light-chain plasmid.

### Recombinant Fab Expression

Human embryonic kidney cells (HEK293-F suspension FreeStyle^TM^ cells; Invitrogen) were transfected with purified plasmid DNA using PEI (Sigma-Aldrich) and cultured in Dulbecco's Modified Eagle medium (DMEM) supplemented with 10% FCS, 2 mmol/L L-glutamine, 10 U penicillin/streptomycin (all Invitrogen) at 37°C and 5% CO_2_. Following selection with 50 ng/ml hygromycin B (Invitrogen), cells were transferred into Freestyle™ 293 expression media (Invitrogen), supplemented with hygromycin B, to spinner culture flasks. Culture supernatants were harvested and filtered.

### Detection of SPE-7 IgE Fab expression and specificity ELISA

SPE-7 IgE Fab protein expression and DNP-specificity was confirmed using ELISA plates (Maxisorp; Nunc) coated overnight with 5 μg/ml DNP-HSA (Sigma-Aldrich), blocked with PBS-1% BSA and incubated with 50 μl cell-free culture supernatants for 2 hours, followed by HRP-conjugated anti-His-tag detection antibody diluted 1/1000 (Novagen). His-tagged Fab was detected with TMB (R&D Systems) by measuring absorbance at 450 nm (Supplementary Figure 2C).

### His-tagged SPE-7 IgE Fab Purification

His-tagged Fab protein was purified by affinity chromatography using a HisTrap FF Crude column and äKTA Prime system (both GE Healthcare). Captured His-tagged protein was eluted with 500 mM imidazole phosphate buffer. Size-exclusion purification was carried out using an HPLC system (Gilson), with Superdex™ 200 column (GE Healthcare) and fractions were collected between 10 and 30 minutes (Supplementary Figure 2D). Purity was estimated by SDS-PAGE analysis (Supplementary Figure 2E).

### Quantification of FcεRI expression by LAD-2 and RBL-2H3 cells

LAD-2 human mast cells (NIH) were isolated from continuous culture in StemPro-34 media with 100 ng/ml stem cell factor (both Invitrogen) or following 5 days priming with 100 ng/ml stem cell factor and 6 ng/ml IL-4 (R&D Systems). RBL-2H3 cells were removed from adherent culture in RPMI supplemented with 10% FCS, 2 mmol/L L-glutamine, 10 U penicillin/streptomycin (all Invitrogen). Cells were incubated with 1 μg primary anti-human or anti-rat FcεRI mouse IgG antibody or isotype control (eBioscience), washed again, and incubated with secondary FITC-conjugated anti-mouse IgG F(ab')_2_ (Qifikit®, Dako). Simultaneously, Qifikit® set-up bead cocktail, a mixture of blank beads and beads bearing a high number of murine IgG monoclonal antibodies, and calibration bead cocktail, a mixture of five populations of beads bearing different, but well-defined numbers of murine IgG monoclonal antibodies, were also incubated with the secondary F(ab')_2_. All incubations were carried out in darkness, at 4°C for 45 minutes. FITC fluorescence was analysed by flow cytometry (FACSCalibur, BD Biosciences).

The set-up bead cocktail was first used to adjust the flow cytometer voltage settings to detect the required range of FITC fluorescence. The median fluorescence intensity (MFI) for each of the five calibration bead populations was determined and plotted against the known number of IgG molecules on the beads to produce a calibration curve. The number of human FcεRI molecules expressed per LAD-2 mast cell was interpolated from the MFI of the stained cell peak using the constructed calibration curve. Flow cytometry data was analysed by FlowJo software (Treestar).

### Mast Cell Activation Assays

RBL-2H3 cells were seeded at 20,000 cells/well in RPMI supplemented with 10% FCS, 2 mmol/L L-glutamine, 10 U penicillin/streptomycin (all Invitrogen) and incubated at 37°C, 5% CO_2_ overnight. Following washing with HBSS, cells were stimulated with 0.5–5 μg/ml SPE-7 IgE (Sigma-Aldrich).

LAD-2 human mast cells (NIH) were primed for 5 days in StemPro-34 media with 100 ng/ml stem cell factor (Invitrogen) and 6 ng/ml IL-4 (R&D Systems) to up-regulate FcεRI expression. In some cases, cells were also sensitised with 3 nM human IgE Fcε_2−4_ (0.2 μg/ml)^47^, non-cytokinergic human IgE/λ 102.1F10 (0.5 μg/ml)^48^, poorly cytokinergic murine IgE/κ 27–74 (0.5 μg/ml; BD Biosciences) or highly cytokinergic murine IgE/λ SPE-7 (Sigma-Aldrich). This sensitising concentration is below the concentration required to activate cells. Cells primed with IL-4 and stem cell factor were resuspended in HBSS, seeded at 80,000 cells/well, and incubated with 30 nM SPE-7 IgE alone, SPE-7 IgE pre-mixed with a 0.33-3333-fold molar excess of monovalent DNP-L-serine (0.01–100 μM; 0.003–27.1 μg/ml; MP Biomedicals LLC), or SPE-7 IgE pre-mixed with 1 - 20-fold molar excess of recombinant SPE-7 IgE Fab (30–600 nM; 5–100 μg/ml) for 4 hours at 37°C. Sensitised cells were removed from culture, resuspended, seeded and incubated with SPE-7 IgE (3, 15 and 30 nM; 0.5, 2.5 and 5 μg/ml), or non-cytokinergic human IgE 102.1F10 or poorly cytokinergic murine IgE 27–74 (both at 30 nM; 5 μg/ml). Cross-linking of IgE-bound FcεRI was carried out in all activation experiments by incubation with 0.1 μg/ml DNP-HSA (Sigma-Aldrich) or 33 μg/ml anti-human IgE antibody (Dako) for 45 minutes at 37°C.

After each incubation, supernatants were harvested (LAD-2 cells were pelleted by centrifugation prior to supernatant removal). Cells were finally lysed with 0.5% Triton X-100 (Sigma-Aldrich) for 30 minutes and the lysates collected. β-hexosaminidase release was measured using 4-methylumbelliferyl N-acetyl-β-D-glucosaminide substrate (Sigma-Aldrich) as previously described^49^ and mast cell degranulation expressed as a percentage of the total for each well. Human TNF-α released into the same cell supernatant was measured using a commercial kit and compared to the standard supplied (Meso Scale Discovery).

### Statistical Analysis

Statistically significant difference between experimental conditions was determined by one-way ANOVA with Dunnett's or Tukey's post-test. All statistical analyses and calculation of IC_50_ values were performed by GraphPad Prism (GraphPad Software, Inc.).

## Author Contributions

H.J.B. and H.J.G. carried out experimental design, H.J.B. performed functional experiments, H.J.B. and H.B. produced the IgE Fab with help from T.S.D., and the manuscript was written by H.J.B., B.J.S. and H.J.G. All authors reviewed the manuscript.

## Supplementary Material

Supplementary Information Supplementary Information
