# Supplementary material for: Mechanism of the Antigen-Independent Cytokinergic SPE-7 IgE Activation of Human Mast Cells in Vitro
Source: Sci Rep. 2015 Apr 20;5:9538. doi: 10.1038/srep09538 (PMC4402612; doi:10.1038/srep09538)
Supplement: Supplementary Information [file srep09538-s1.pdf]

Mechanism of the Antigen-Independent Cytokinergic SPE-7 IgE Activation of Human Mast Cells *in Vitro*

Heather J Bax, Holly Bowen, Tihomir S Dodev, Brian J Sutton, Hannah J Gould

Supplementary Information:

Supplementary Table – Primers

Supplementary Figure 1 – SPE-7 IgE Fab Amino Acid Sequence

Supplementary Figure 2 – SPE-7 IgE Fab Cloning, Expression and Purification

Supplementary Table - Primers

Where primers were used to add a His-Tag or homologous ends, these regions are underlined.

Primer	Sequence
SPE-7 IgE Heavy and light chain amplification from NS1 hybridoma cells	
SPE-7_L_F	CAGGCTGTTGTGACTCAGGAATC
SPE-7_L_R	CTAGGAACAGTCAGCACGGGA
SPE-7_H_F	GAGGTGCAGCTGCAGCAG
SPE-7_H_R	CTAGGAGGGACGGAGGGAG
SPE-7 IgE Fab heavy chain amplification and His-Tag addition for PIPE cloning	
Fab_H_R1_F	GCGAATTCGCCACCATGGACT
Fab_H_R1_R	CTAGTGATGGTGATGGTGATGGACAGGTCGAACTAGGAT
Fab_H_R2_F	CGCTAATTCAAAGCAAATGGACTGGACCTGGAGGATCCTC
Fab_H_R2_R	ATGTCTGGCCAGCTAGCTAGTGATGGTGATGGTGATGGAC
SPE-7 IgE Light chain amplification for PIPE cloning	
BspEI_L_F	GCTAATTCAAAGCAATATGTTGCCATCACAACTCATTGGG
AvrII_L_R	GGAAACCTGCTCCTAGCTAGACTAGGAACAGTCAGCACGG

SPE-7 IgE Fab Heavy Chain

EVQLQQPGAE LVKPGASVKL SCKASGYTFT SYWMHWVKQR PGRGLEWIGR

SPE-7 VH

IDPNGGGTKY NEKFKSKATL TVDKPSSTAY MQLSSLTSED SAVYYCARMW

YYGTYYFDYW GQGTTLTVSS ASIRNPQLYP LKPCKGTASM TLGCLVKDYF

Murine C ε1

PGPVTVTWYS DSLNMSTVNF PALGSELKVT TSQVTSWGKS AKNFTCHVTH

PPSFNESRTI LVRPV

SPE-7 IgE Fab Light Chain

QAVVTQESAL TTSPGETVTL TCRSSTGAVT TSNYANWVQE KPDHLFTGLI

SPE-7 VL

GGTNNRAPGV PARFSGSLIG DKAALTITGA QTEDEAIYFC ALWYSNHLVF

GGGTKLTVLG QPKSSPSVTL FPPSSEELET NKATLVCTIT DFYPGVVTVD

Murine C λ

WKVDGTPVTQ GMETTQPSKQ SNNKYMASSY LTLTAGAWER HNSYSCQVTH

EGHTVEKSLS RADCS

Supplementary Figure 1– SPE-7 IgE Fab Amino Acid Sequence
SPE-7 IgE Fab heavy and light chain amino acid sequences (GenBank Accession Nos. KJ734989 and KJ734990), derived from NS1 hybridoma cells, and cloned and expressed as SPE-7 IgE Fab protein.

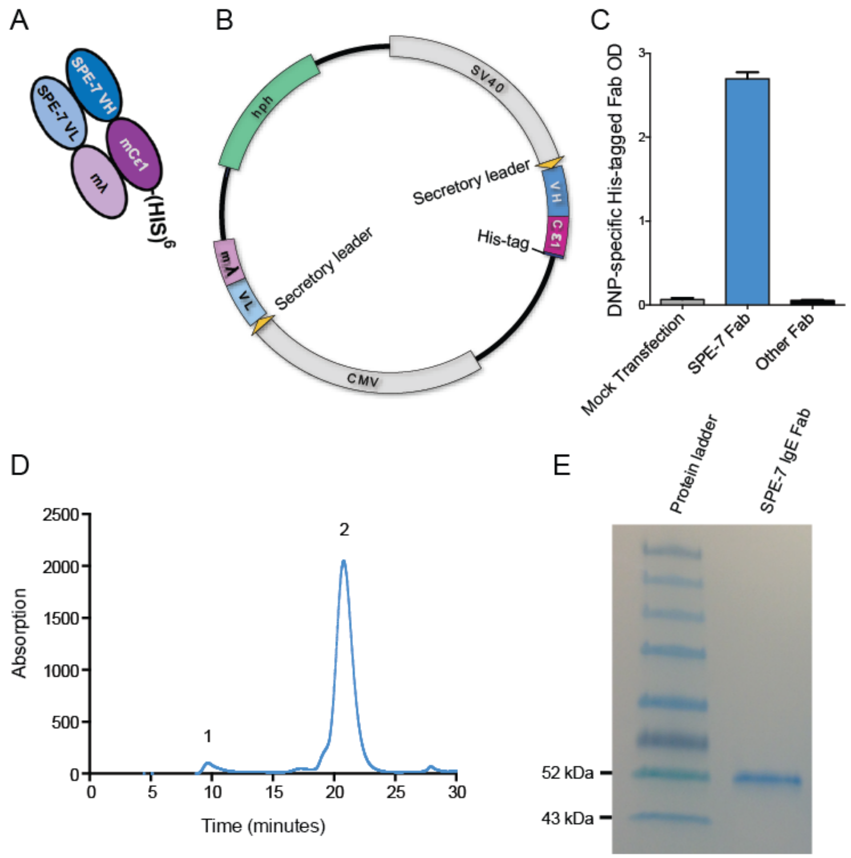

Supplementary Figure 2 – SPE-7 IgE Fab Cloning, Expression and Purification
Schematic of the SPE-7 IgE Fab construct (A) and pVITRO1 vector containing IgE Fab heavy chain (VH and Cε1) and light chain (VL and CL) used to transfect HEK293F cells for mammalian expression (B). Anti-his-tag ELISA to measure expression of DNP-specific SPE-7 IgE Fab by HEK293F cells (C). Size-exclusion chromatography purification profile (D). Monomeric IgE Fab was eluted at around 20 minutes (2) and these fractions collected to remove aggregated protein (1). SDS-PAGE analysis of SPE-7 IgE Fab protein under non-reducing conditions (E).
